# The Effect of the Irreversible Inequality on Pro-social Behaviors of People With Disabilities

**DOI:** 10.3389/fpsyg.2019.00012

**Published:** 2019-01-31

**Authors:** Shen Liu, Zhongchen Mou, Wenlan Xie, Chong Zhang, Yijun Chen, Wen Guo, Xiaochu Zhang, Lin Zhang

**Affiliations:** ^1^Department and Institute of Psychology, Ningbo University, Ningbo, China; ^2^School of Humanities and Social Science, University of Science and Technology of China, Hefei, China; ^3^School of Psychology, Nanjing Normal University Nanjing, China; ^4^Ningbo Institute of Education, Ningbo, China; ^5^Department of Mechanical and Automation Engineering, The Chinese University of Hong Kong, Hong Kong, China; ^6^CAS Key Laboratory of Brain Function and Disease, School of Life Sciences, University of Science and Technology of China, Hefei, China; ^7^Hefei Medical Research Center on Alcohol Addiction, Anhui Mental Health Center, Hefei, China; ^8^Academy of Psychology and Behavior, Tianjin Normal University, Tianjin, China

**Keywords:** irreversible inequality, pro-social behaviors, reciprocity, people with disabilities, discrimination

## Abstract

Inequalities have always been central to psychology, sociology and related fields such as social policy, gender studies, critical race studies, and human geography. Although inequality affects pro-social behaviors, there are still some controversies over this issue among people with disabilities. The current study aimed to investigate pro-social behaviors of people with disabilities and the effect of the irreversible inequality on pro-social behaviors. A dictator game was used to explore the difference of pro-social behaviors between people with disabilities and people without disabilities, when facing intra- or inter-group members. The results showed that compared to people with disabilities, people without disabilities were likely to show more pro-social behaviors. People with disabilities preferred intra-group cooperation, while people without disabilities preferred inter-group cooperation. Indeed, the intra-group cooperation was significantly greater than the expected cooperation of the intra-group members for people with disabilities. When facing the inter-group members, people without disabilities showed more than expected, that others would cooperate with them. These findings indicated that social avoidance was a common phenomenon for people with disabilities in China, but the situation would be different when they faced different groups. In addition, irreversible inequality could influence individuals’ cooperative strategies when facing individuals in a different status.

## Introduction

Inequality has always been central to psychology, sociology and related fields such as social policy, gender studies, critical race studies and human geography. There is evidence that people with disabilities have lower social capital than people without disabilities (Mithen et al., 2015). Many studies have shown that reversible inequality (e.g., wealth, power, and status) influences pro-social behaviors (Han et al., 2009; Rao et al., 2011; Caprara et al., 2012; Inesi et al., 2012; Lammers et al., 2012; Cai et al., 2016). However, there are still some controversies over the issue of irreversible inequality among people with disabilities. Some studies show that compared to disadvantaged people (low-power individuals), people in a dominant position (high-power individuals) always show more selfishness, hindering their understanding of others’ emotions, keeps them away from others, and inhibits their pro-social behaviors (Lammers and Stapel, 2011; Gwinn et al., 2013; Magee and Smith, 2013). Other studies demonstrate that individuals in a dominant position always show more pro-social behaviors due to the evoked altruistic traits and enhanced control of valuable resources, than those in a dominated position (Han et al., 2009; Kraus et al., 2011; Rao et al., 2011; DeCelles et al., 2012; Williams, 2014; Liu et al., 2018), and disadvantaged people always show more pro-social behaviors toward others, than advantaged people do (Han et al., 2009; Rao et al., 2011). Han et al. (2009) showed that pre-school children playing in their own classroom made fewer offers (cookies) in the game than those cooperating with others. Liu et al. (2018) found that disabled people preferred to interact with other disabled people and had higher cooperation, satisfaction and sense of justice when interacting with disabled people than when interacting with abled people. It is considered that pro-social behaviors of humans also differ when they face individuals in different status groups (intra- or inter-group).

However, in previous studies, the status of people with disabilities was mostly temporary or reversible in experiments. Rao et al. (2011) revealed that the degree of pro-social behaviors increased with an increasing level of residential devastation, but decreased with the passage of time. Moreover, with the improvement of the disadvantageous status, their pro-social behaviors gradually reduced. Then, if the advantageous and disadvantageous status is irreversible or stable in the long term, will irreversible inequality affect pro-social behaviors? Some studies show that when advantaged people believed others couldn’t be any potential threat to their status, they tended to allocate more resources and showed more pro-social behaviors to the inferior, for a perceived sense of social responsibility (Handgraaf et al., 2008). Thus, the current study hypothesized that people with an irreversible advantage showed more pro-social behaviors than people with a disadvantage. Therefore, it’s necessary to explore the pro-social behaviors of people with disabilities and people without disabilities, when they face intra- and inter-group members and its underlying mechanism.

The mechanism of pro-social behavior is a hot topic in the field of social psychology. Researchers have put forward various theories and models to explain pro-social behaviors, such as the kin selection, the group selection and the reciprocation (Jones, 2018). The group select, also called the group selection theory, suggests that individuals are more willing to help members in the same group (de Dreu et al., 2010). Pro-social behaviors occurring between strangers can be explained by the reciprocity theory. The early reciprocal theory suggested that people showed pro-social behaviors for the purpose of benefitting the individual, and fundamentally emphasized the self-interest tendency of pro-social behaviors, which is a kind of direct or indirect reciprocity (Trivers, 1971; Axelrod and Hamilton, 1997). However, the late strong reciprocity theory focuses on the individuals’ own existence. This theory proposes that pro-social behaviors are not an act out of the tendency of self-interest, but one’s better survival (Gintis et al., 2003; West et al., 2007; Xie et al., 2013). Therefore, the mechanisms for showing pro-social behaviors may be different when individuals face intra- and inter-group members.

Different from previous studies, the current study selected people with disabilities as individuals at the irreversible disadvantage. Compared with people without disabilities, the physical disabilities of people with disabilities are difficult to change. Their employment rate and salaries are also lower than abled people, even though the government has passed laws to protect their interests (Deleire, 2000). In addition, most people with disabilities reported discrimination and unfair treatment from people without disabilities (Moore et al., 2011). Because of the existence of discrimination, people with disabilities often question their own abilities, feel inferior to people without disabilities and have low self-esteem, which can lead to serious psychological and social adaptation problems (Santuzzi, 2011). In this study, unfairness is an irreversible and long-term stable factor. Thus, it can be considered that people with disabilities are at a irreversible and stable disadvantage. This study explores the differences and mechanisms of pro-social behaviors between disadvantaged people and advantaged people, when facing intra- and inter-group members, mainly according to the Group Selection Theory.

From the discussion above, the current study explored the following three issues: (a) The pro-social behaviors of people with disabilities and people without disabilities; (b) and how irreversible inequality affected their pro-social behaviors. In other words, what was the difference between pro-social behaviors of people with disabilities and people without disabilities when they faced intra- and inter-group members? And (c) The mechanism of pro-social behaviors of people with disabilities and people without disabilities when they faced intra- and inter-group members.

## Materials and Methods

### Participants

A total of 102 residents in the Zhejiang province in China were recruited, including 47 participants with disabilities (30 males, 17 females) with an average age of 46.8 years (*SD* = 6.1), and 55 participants without disabilities (20 males, 35 females) with an average age of 48.2 years (*SD* = 8.3). Participants with disabilities were of normal intelligence and had a PRC Certificate of Disabled Person. All individuals were measured one-to-one. The entire experiment process completely followed the voluntary principle. A small gift was offered in return for participation. The Ethics Committee of Ningbo University approved this study, in accordance with the ethical principles of the Declaration of Helsinki.

### Dictator Game and Expectation Game

Behavioral economists have used dictator games for over two decades to study pro-social behavior (Hoffman et al., 1994). This game is a particularly interesting way to test generosity and pro-social behavior, because it is an asymmetric game that the recipient is obliged to accept the sum offered by the dictator. Because the dictator does not have to fear the rejection of its proposal, as in the ultimatum game, the motivations behind a dictator’s behavior are assumed to be free of strategic considerations (Thunström et al., 2016; Ibanez et al., 2017; Miklánek, 2018; Thielmann and Hilbig, 2018). In the most common version of the game, the dictator receives an initial endowment of $10 and is asked what amount he is willing to share with an anonymous co-player (Schier et al., 2016). The current study modified the traditional dictator game, distinguished the participants by types (intra-group and inter-group) with whom the participants were going to cooperate, and increased the expectations of others pro-social behaviors, to investigate the attitudes and behaviors of people with disabilities and people without disabilities, when they faced intra-or inter-group members. The experiments were single blind. The detailed materials are described in the following section.

DICTATOR GAME:

Suppose now you are provided with ¥100 in cash and are asked to propose a division of ¥100 between yourself and an anonymous person:

If the anonymous person has a disability, you will offer ¥_____ to him.
If the anonymous person does not have a disability, you will offer ¥_____ to him.

EXPECTATION GAME:

Suppose now that an anonymous person is provided with ¥100 in cash and is asked to propose a division of ¥100 between themselves and you:

If the anonymous person has a disability, you expect that you will be offered ¥_____.
*If the anonymous person does not have a disability, you expect that you will be offered ¥_____*.

In the Dictator Game, the amount offered by the recipient was used as the measure of actual pro-social behaviors. In the Expectation Game, the amount offered by the recipient was considered as the expectation of pro-social behaviors of others.

### Design and Data Analysis

A 2 (types of participants: people with disabilities/people without disabilities) × 2 (types of tasks: cooperation/expectation) × 2 (types of groups: intra-group/inter-group) ANOVA was used to the types of participants as the between-subject factor, and types of tasks and types of groups as the within-subject factors. The dependent variable was the amount of money offered by the participants. The mean money offered by participates were collated and calculated with SPSS 19.0, and the significance level was set at *p* < 0.05.

## Results

### Pro-social Behaviors of People With Disabilities and People Without Disabilities

An independent sample *t*-test was conducted to measure the difference between the pro-social behaviors of people with disabilities and people without disabilities. Results indicated a significant difference (*t*_100_ = -2.675, *p* < 0.01, *d* = -0.62). People with disabilities (*M* = 84.35, *SD* = 51.60) showed fewer pro-social behaviors than people without disabilities (*M* = 112.91, *SD* = 38.95). In other words, compared to people with disabilities, people without disabilities were likely to show more pro-social behaviors.

### Pro-social Behaviors of People With Disabilities and People Without Disabilities When They Faced Intra- and Inter-Group Members

In order to investigate pro-social behaviors of people with disabilities and people without disabilities when they faced intra- and inter-group members, a 2 (types of participants: people with disabilities/people without disabilities) × 2 (types of tasks: cooperation/expectation) × 2 (types of groups: intra-group/inter-group) ANOVA was used, with the types of participants as the between-subject factor and the types of tasks and types of groups as the within-subject factor. Results indicated a significant main effect of types of tasks [*F*(1,100) = 19.18, *p* < 0.001, $\eta_{p}^{2}$ = 0.213], and no significant main effect of the types of participants was found [*F*(1,100) = 3.11, *p* = 0.082]. In addition, the main effect of the types of groups also reported no significant difference [*F*(1,100) = 0.408, *p* = 0.525].

**FIGURE 1:**
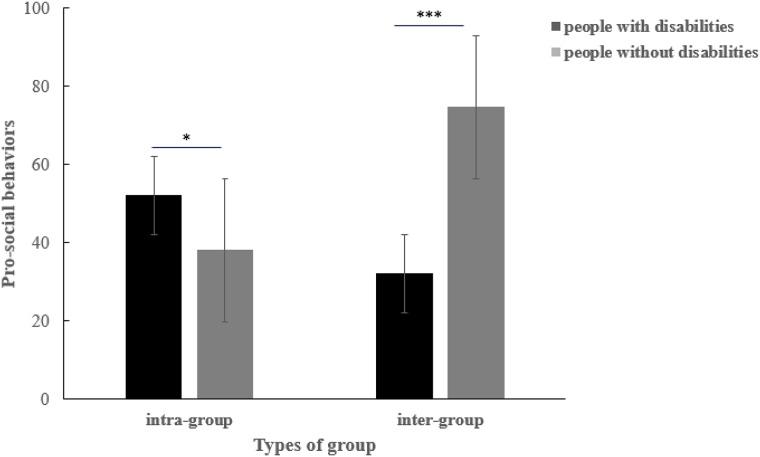
The pro-social behaviors of people with disabilities and people without disabilities, when they faced members of a different group. ^∗^*p* < 0.05, ^∗∗^*p* < 0.01, ^∗∗∗^*p* < 0.001, one-tailed.

There was an interaction between types of participants and types of tasks [*F*(1,100) = 33.71, *p* < 0.001, $\eta_{p}^{2}$ = 0.322]. An interactive analysis was conducted (see Figure 1) in order to investigate the difference between pro-social behaviors of people with disabilities and people without disabilities, when they faced the intra- and inter-group members. Compared to people without disabilities (*M* = 38.18, *SD* = 2.72), people with disabilities (*M* = 52.17, *SD* = 4.21) showed more pro-social behaviors when they faced intra-group members [*F*(1,100) = 7.78, *p* < 0.01, $\eta_{p}^{2}$ = 0.093]. However, people with disabilities (*M* = 32.17, *SD* = 6.01) showed fewer pro-social behaviors than people without disabilities [*M* = 74.73, *SD* = 3.93; *F*(1,100) = 34.57, *p* < 0.001, $\eta_{p}^{2}$ = 0.313] when they faced inter-group members. Namely, people with disabilities preferred intra-group cooperation while people without disabilities preferred inter-group cooperation.

There were interactions between types of participants and types of groups [*F*(1,100) = 6.30, *p* < 0.01, $\eta_{p}^{2}$ = 0.082], types of tasks and types of groups [*F*(1,100) = 5.13, *p* < 0.05, $\eta_{p}^{2}$ = 0.067], and there was a three-way interaction between types of participants and types of tasks [*F*(1,100) = 16.96, *p <* 0.001, $\eta_{p}^{2}$ = 0.193]. To break up the three-way interaction, the current study analyzed pro-social behaviors and the expectation of people with disabilities and people without disabilities, respectively.

**FIGURE 2:**
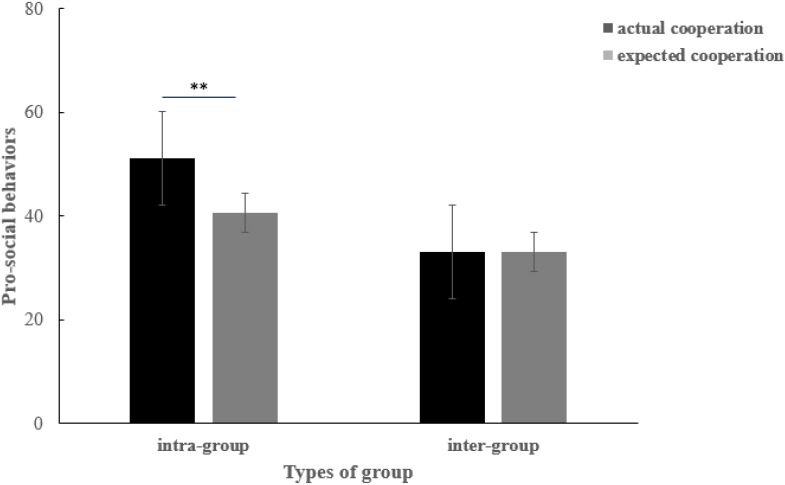
The actual and expected cooperation of people with disabilities when they faced intra-group and inter-group members, respectively. ^∗^*p* < 0.05, ^∗∗^*p* < 0.01, ^∗∗∗^*p* < 0.001, one-tailed.

For people with disabilities, a 2 (types of tasks: intra-group/inter-group) × 2 (types of groups: intra-group/inter-group) within-subject ANOVA was conducted. Results indicated a significant main effect of types of tasks [*F*(1,46) = 9.38, *p* < 0.01, $\eta_{p}^{2}$ = 0.056], no significant main effect of types of groups [*F*(1,46) = 1.01, *p* = 0.33], and no significant interaction [*F*(1,46) = 1.14, *p* = 0.30]. A paired sample *t*-test (see Figure 2) showed that intra-group cooperation (*M* = 51.11, *SD* = 4.64) was significantly greater than the expected cooperation (*M* = 40.56, *SD* = 7.29) of the intra-group members (*t*_100_ = 2.20, *p* < 0.01, *d* = 1.71).

For people without disabilities, a 2 (types of tasks: intra-group/inter-group) × 2 (types of groups: intra-group/inter-group) within-subject ANOVA was conducted. Results indicated a significant main effect of types of tasks [*F*(1,54) = 41.38, *p* < 0.001, $\eta_{p}^{2}$ = 0.43], a significant main effect of types of groups [*F*(1,54) = 50.56, *p* < 0.001, $\eta_{p}^{2}$ = 0.48], and a significant interaction [*F*(1,54) = 41.31, *p* < 0.001, $\eta_{p}^{2}$ = 0.433].

**FIGURE 3:**
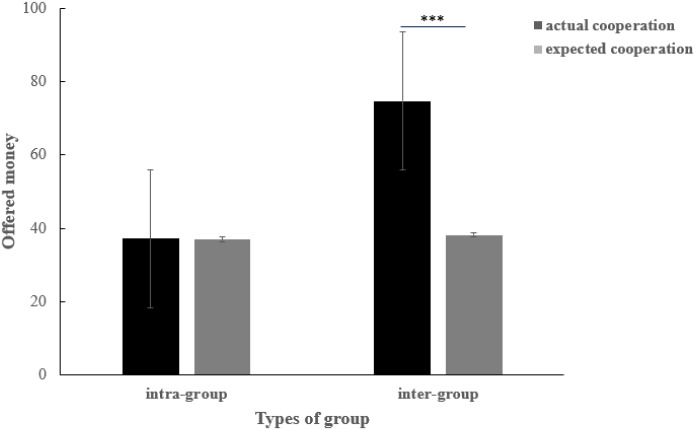
The actual and expected cooperation of people with disabilities and people without disabilities, when they faced members of the intra-group and inter-group, respectively. ^∗^*p* < 0.05, ^∗∗^*p* < 0.01, ^∗∗∗^*p* < 0.001, one-tailed.

An interactive analysis was conducted and results are illustrated in Figure 3. When facing inter-group members, people without disabilities offered more (*M* = 74.73, *SD* = 3.68) than they expected others would cooperate with them [*M* = 37.10, *SD* = 3.18; *F*(1,54) = 49.27, *p* < 0.001, $\eta_{p}^{2}$ = 0.477]. In addition, there was no significant difference between expectation (*M* = 36.91, *SD* = 3.16) and real cooperation (*M* = 38.18, *SD* = 2.70) when they faced members in intra-groups [*F*(1,54) = 0.73, *p* = 0.397].

**FIGURE 4:**
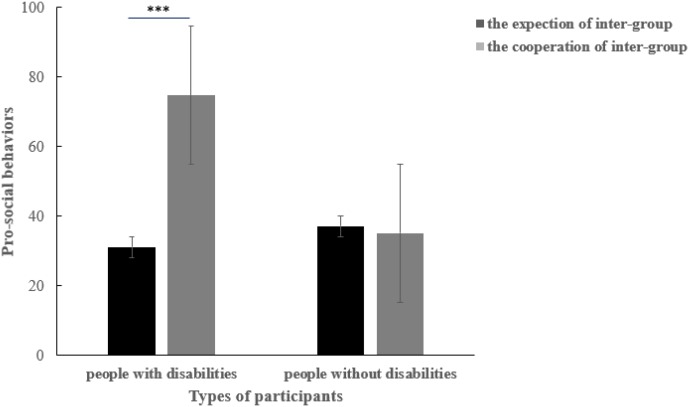
The offered and expected cooperation of people with disabilities and people without disabilities, when they faced inter-group members respectively. ^∗^*p* < 0.05, ^∗∗^*p* < 0.01, ^∗∗∗^*p* < 0.001, one-tailed.

A further analysis was also conducted between the expected cooperation of people with disabilities and the actual cooperation of people without disabilities, as well as the expectation cooperation of people without disabilities and the real cooperation of people with disabilities. Surprisingly, results demonstrated that the former showed a significant difference (*t*_100_ = -6.04, *p* < 0.001, *d* = 1.21), and the latter showed no significant effect (*t*_100_ = 0.176, *p* = 0.861). It indicated that people with disabilities might have a misunderstanding of people without disabilities, showing more cooperation (*M* = 74.73, *SD* = 27.24) than people with disabilities expected (*M* = 31.11, *SD* = 28.41). However, the expectations of people without disabilities (*M* = 37.11, *SD* = 23.85) were consistent with the real cooperation of people with disabilities (*M* = 35.11, *SD* = 30.85; see Figure 4).

## Discussion

The current study aims to investigate the effect of irreversible inequality on cooperation or pro-social behaviors. Compared to people without disabilities, people with disabilities were selected as individuals with an irreversible inequality status, to explore the difference of pro-social behaviors when they faced intra- or inter-group members and its underlying mechanism. Results indicated that people without disabilities were likely to show more pro-social behaviors compared to people with disabilities. It was consistent with previous studies (Kraus et al., 2011; DeCelles et al., 2012; Williams, 2014). In general, people without disabilities had higher cooperation compared to people with disabilities.

However, when individuals faced members in a different status, results were different. Results showed that people with disabilities preferred intra-group cooperation, while people without disabilities preferred inter group cooperation. According to previous studies, when people without disabilities believed others couldn’t pose any potential threat on their status, they tended to allocate more resources and showed more pro-social behaviors to the inferior, for a perceived sense of social responsibility (Handgraaf et al., 2008). When they faced individuals of the same status, the sense of responsibility might decline. For people with disabilities, the mutual aid could serve as an adaptive mechanism to increase the individual’s survival opportunities (Rao et al., 2011), so they would show more pro-social behaviors when they faced individuals of the same status. However, studies have shown that most people with disabilities reported discrimination and unfair treatment from people without disabilities (Moore et al., 2011), therefore the long-term disadvantageous position of people with disabilities would eventually lead to their misunderstanding of people without disabilities. In the current study, people with disabilities offered less to the advantaged, which might be due to the perceived discrimination.

It is also important to discuss the mechanisms of showing pro-social behaviors, when people with disabilities and people without disabilities faced individuals of a different status. When people without disabilities faced inter-group members, they offered more than they expected others would cooperate with them. On the contrary, when people with disabilities faced intra-group members, they offered more than they expected others would cooperate with them. Strong reciprocity might play a role in both cases (Gintis et al., 2003; West et al., 2007; Xie et al., 2013). There was no significant difference between expectation and real cooperation when people without disabilities were in intra-groups and people with disabilities were in inter-groups. Direct or indirect reciprocity might occur (Trivers, 1971; Axelrod and Hamilton, 1997). Therefore, the irreversible inequality could influence the cooperative strategies of people without disabilities when they faced individuals of a different status.

Overall, the results provided another perspective of cooperation between individuals in the irreversible inequality status. When individuals have a different status but are not in a competitive circumstance, “in-group favoritism”— e.g., individuals offer more cooperation to the intra-group than to the inter-group (Masuda, 2012), but to some extent, this may not always be the case. Individuals in irreversible dominance were against the “intra-group favoritism,” and offered more cooperation, which supports that individuals with an advantage would provide more help (Han et al., 2009; Kraus et al., 2011; Rao et al., 2011; DeCelles et al., 2012; Williams, 2014). In the current study, when they faced inter-group members, people without disabilities offered more than they expected others would cooperate with them. In this circumstance, strong reciprocity might occur, and the effect was much stronger than “favor.” Or it could be a performance of inequality-averse social preferences. For example, Tricomi et al. (2010) found that those with an advantage (high-pay) status preferred monetary transfer that reduced inequality. In the current study, people without disabilities possessed a sane body, just like the high-pay participants with an advantage status.

On the contrary, there is a difference for those with an irreversible disadvantaged status. There might be two reasons: (i) they do not distinguish themselves from others, or (ii) they may misunderstand the justice of the distribution when they face different group members. The first assumption has been denied due to the groups’ apparent difference, and showed more cooperation than those with an advantageous status, when status was analyzed as a cooperation target, respectively. It indicated that the inferior showed more cooperative willingness in same status than the superior. This finding, as an index, was congruent with the theory that a disadvantage made people more cooperative to some extent. The second assumption might be reflected by the average amount of money offered by people with disabilities: ¥52.17 and ¥32.17 (the former for people with disabilities and the latter for people without disabilities) and the average of the actual amount of money offered by people without disabilities (*M* = 74.73) was more than the expected average amount for people without disabilities (*M* = 31.11).

In addition, individuals’ expectation does not always conform to cooperation offered by others. An noteworthy finding was that there was a big misunderstanding of the recognition of people with disabilities between their expectancy and others’ offerings. Our findings were consistent with previous studies. In addition, it should be pointed out that the inferior considered individuals in the same status and gave them more help than those in a different status. However, those at the advantage showed no difference between the same status and the different status. It could also be supposed that the inferior had a bias toward others. Taking people with disabilities into account, the perceived stigma might be considered. From another point of view, the fact that individuals at the advantage offered more help to the inferior could be regarded as a source of stigma. In Chen and Shu’s (2012) study, they found that abled students provided with additional support through handicapped identity cards, also demonstrated a source of stigma.

The current study has some implications. Firstly, the government should promote the social security and service system of people with disabilities, especially by improving their social status and value, not just raising their income. Promoting the social status of people with disabilities may reduce their social avoidance; subsequently, alleviating negative emotions such as anxiety and depression. It is imperative to eliminate others’ discrimination toward people with disabilities as well as their self-discrimination, through the use of mass media or through support from other people. The discrimination that those with disabilities perceive stems from self-reports, which is non-existent. The public and government can use public services to advertise and provide assistance to eliminate discrimination, which is perceived as wrong, and people without disabilities should be educated on how to communicate and assist people with disabilities in correct and considered ways. Last but not the least, cognitive training services to improve the negative cognitive bias (such as attentional and interpretational bias toward social cues) toward people with disabilities are also needed, as some studies have shown that people with disabilities have attentional sensitivities to negative social emotions, and greater negative interpretational bias to the ambiguous social cues (Zhang et al., 2014, 2015; Mou et al., 2017). Therefore, people with disabilities also misunderstand others’ intentions. Based on the aforementioned findings, the current study encourages methods that can be adopted to intervene with attentional and interpretational processing characteristics, such as specialized attentional training which by repeatedly presenting positive and pleasant external stimuli, gradually improves the habitual attention and interpretation of people with disabilities to process tendency, thereby, eliminating others’ discrimination and promoting pro-social behaviors, improving their physical and mental health.

However, there are still some limitations of the current study that need to be improved in future studies. First, it should be noted that the current study used the non-repeated game and corresponding results may be unstable. Second, although people with disabilities are the vulnerable groups, they cannot represent all groups at a disadvantage. Therefore, the social exchange process and other types of participant selection need to be considered in future studies. What’s more, the study did not take the effect of environmental factors on pro-social behaviors into consideration, for example, a competitive condition can be set to explore the mechanism of reciprocity and “in-group favoritism.”

## Conclusion

The current study found that compared to people with disabilities, people without disabilities were likely to show more pro-social behaviors. People with disabilities preferred intra-group cooperation, while people without disabilities preferred inter-group cooperation. For the people with disabilities, intra-group cooperation was significantly greater than the expected cooperation of intra-group members. When facing inter-group members, people without disabilities offered more than they expected others would cooperate with them. It indicated that social avoidance was a common phenomenon for people with disabilities in China.

## Author Contributions

SL, XZ, and LZ conceived and writing frame design. SL, ZM, WX, CZ, YC, and WG wrote the paper. SL, ZM, WX, CZ, YC, WG, XZ, and LZ revised the manuscript.

## Conflict of Interest Statement

The authors declare that the research was conducted in the absence of any commercial or financial relationships that could be construed as a potential conflict of interest.
